# Metrorrhagia

**Published:** 1889-03

**Authors:** P. R. Cortelyou

**Affiliations:** Georgia


### METRORRHAGIA.

BY P. R. CORTELYOU, M. D., OF GEORGIA.

Metrorrhagia, a profuse and excessive flow of blood at the menstrual periods, we recognize as menorrhagia, whlie in Metrorrhagia, the loss of blood is not confined to the menstrual periods, but many occur at any time.

This latter trouble, as well as the former, is of frequent occurrence, and is caused by a large number of affections, functional and organic.

Any thing which produces active or passive congestion of the uterus, will act as an exciting cause. Also anything which may cause an abrasion of the mucous membranes, or any foreign growths in the body or neck of the uterus may be the cause of the excessive hæmorrhage from that organ.

At the period of the menopaus congestion and free hæmorrhage are of frequent occurrence.

Retained placenta, after abortion, miscarriage; also fungous growths and hydatids, cause the same trouble. Constipation, and impacted foecal matter will also at times produce the same results.

Hæmorrhage then from the uterine canal, is but a symptom which may be due to many different causes; therefore it becomes necessary, in these cases instead of treating symptoms only, to examine systematically the organ and so endeavor to find the exact cause of the hæmorrhage.

This examination should be digital, and with the speculum, and uterine sound; the whole uterine canal being dilated if necessary, so that the whole interior of the uterus may be fully explored.

One reason why failure to cure is so frequent, is because the prominent symptom, hæmorrhage, is treated, and the disease which is the cause is not cured.

Some of the results that follow from Metrorrhagia, if not relieved by appropriate treatment are: sterility, neurasthenia, dyspepsia, hydraemia, death.

The two following cases which came under my care, are reported as showing the results secured from a certain line of treatment:

Case 1. Mrs.—aged 39 years, dark complexion, nervous temperament, never had any children, consulted by the patient Nov. 27, 1888. She gave the following history: Had been troubled several times during past three years with excessive flowing, her menstrual period being very irregular. Had also suffered with leucorrhæl discharge, and heat and burning of vagina. During the summer she had an attack of Remittent fever.

About ten days previous to my being consulted, she commenced to have some flooding, which gradually increased, becoming very profuse. She had used douches of hot water, persulphate of iron, and fluid ext. of ergot internally. Although on former occasions these measures had controlled the hæmorrhage they now fail to do so.

There being no hæmorrhage at the time of my visit I added fluid ext. black haw in ½ʒ doses 4 or 5 times a day, rest on the back, cool drinks and cold cloths to vulva and over the uterus if bleeding returned. Within twenty-four hours hæmorrhage returned with increased violence. I then washed out the vagina which was filled with hard clots from the alum and iron injections, and introducing the speculum discovered the blood flowing freely from the mouth of the uterus.

The uterus was much enlarged and retroflexed, the fundus pressing against the rectum. Applied liq ferri persulphate freely to the neck, and plugged with absorbent cotton wet with same, then tamponed the vagina. About eighteen hours after making this application, found hæmorrhage still uncontrolled; so removed tampon, and used hot water douche freely, then made same treatment Nov. 30, 1888. The hæmorrhage still continuing and the patient becoming very weak, and nervous from continual loss of blood, I called Dr. G. G. Roy of Atlanta in consultation. Upon examination he concluded the case to be one of fungous growths of the mucous membrane lining the cavity of the uterus.

He made a very thorough application to the internal surface of the uterus of a solution of carbolic acid, glycerine and iodine equal parts. This checked the bleeding for about 18 hours, when it returned, and I then made the same treatment. The case continued this way for several days before the bleeding was completely checked.

The solution was applied every two or three days after bleeding had been checked until a free discharge occurred; and the patient rapidly gained strength, on nourishing diet and tonics. She was urged to allow the uterus to be thoroughly curetted, but being anxious to return to her home in Florida, decided to wait and see how she would be in the future.

Case 2. Called Dec. 9, '88 to see Mrs. G., aged about 30 years, no children. Had been flowing freely for nearly three weeks. Gave history of similar trouble in the past. Menstruation had been very irregular for several years. She was weak and anæmic from loss of blood. On examination with speculum found the neck of the womb very much congested, with erosions at the os and the blood coming freely from the uterine canal.

I applied the carbolic iodine and glycerine solution freely to internal surface of the neck and body of the uterus, which checked the hemorrhage. I then applied a tampon of absorbent cotton filled with tannic acid to the os, and allowed it to remain in situ for twenty-four hours.

After removal, bleeding returned, but not so freely. Made the same applications, and from this time bleeding ceased.

The glycerine, iodine and carbolic acid preparation was then applied twice a week until there was a free discharge from the uterus, and the organ had resumed a healthy appearance. The patient during local treatment was given liberal diet and tonics of iron, quinine and strychnine. She rapidly recovered her strength, and has been in good health since that time.

These cases, with others which might be added, are reported to show the good results following the use of the carbolic acid, glycerine and iodine solution, and the necessity in cases of the kind of making a free and thorough application directly to the diseased parts.

